# The Effect of Concerns About COVID-19 on Anxiety, Stress, Parental Burnout, and Emotion Regulation: The Role of Susceptibility to Digital Emotion Contagion

**DOI:** 10.3389/fpubh.2020.567250

**Published:** 2020-12-18

**Authors:** Alena Prikhidko, Haiying Long, Michael G. Wheaton

**Affiliations:** ^1^Department of Counseling, Recreation and School Psychology, Florida International University, Miami, FL, United States; ^2^Department of Educational Psychology, University of Kansas, Lawrence, KS, United States; ^3^Barnard College, Columbia University, New York, NY, United States

**Keywords:** COVID-19, parental burnout, concern about COVID-19, digital emotion contagion, emotion regulation

## Abstract

**Background and aims:** The COVID-19 pandemic has caused social and economic turmoil, which has led to enormous strain for many families. Past work with pandemic outbreaks suggests that media attention can increase anxiety and compensatory behaviors. Social isolation can lead to increase in online communication and parents who use social media may be affected by other people's emotions online through what is known as digital emotion contagion (DEC). The current study aimed to examine the role of DEC in the relationship between stress, concern about COVID-19, parental burnout and emotion regulation (ER).

**Methods:** In April 2020, an online survey was advertised in Social Media Parenting Groups and published on FIU Psychology online research system SONA. Data were analyzed using correlational analysis, linear and multiple linear regression, and moderation analysis.

**Results:** Concern about COVID-19 predicted stress, depression, and parental burnout. Susceptibility to DEC significantly increased the impact of stress on parental burnout. Having relatives infected with COVID-19 increased the effect of DEC on parental burnout. A higher level of ER buffered the relationship between emotion contagion and concern about COVID-19.

**Conclusion:** These findings suggest that susceptibility to digital emotion contagion may have a negative effect on parents. Digital emotion contagion may increase parental burnout and is tied to stress.

## Introduction

The COVID-19 pandemic has caused social and economic turmoil, which has led to enormous strain for many families (1, 2). Parents who are currently living with children may have been particularly impacted by COVID-19 due to social and physical isolation, the risk of unemployment, the financial strain, and the challenges of balancing work and family life while schools are on lockdown. Increasing numbers of US mothers have symptoms of clinical depression and anxiety during the pandemic (3, 4). Anxiety may lead to burnout (5), which can have detrimental consequences for parents' and children's well-being (6). It is important to study the ways that American families have been affected by the COVID-19 outbreak to determine how concerns about or fears of the virus may have led to excessive anxiety, stress, and parental burnout and to understand whether parental emotion regulation (ER) strategies may have buffered these effects.

In addition, being on lockdown during quarantine, many parents and children have used telecommunication to stay connected with their family, friends, teachers, and colleagues. The majority of contact occurs via the internet, which was actively used by mothers before the Pandemic: during the year 2018, 59% of U.S. mothers accessed social media several times per day, and spent 214 min browsing the internet on a daily basis (7). Thus, it is worth considering the ways in which digital connections may have affected mental health. As it spreads, COVID-19 has been frequently reported in mass media and social media (e.g., Facebook and Twitter). Importantly, past work with pandemic outbreaks suggests that media attention can increase anxiety and compensatory behaviors (8). Online communication takes a multitude of forms, including navigating social media either actively (e.g., posting on Facebook parenting groups and asking for advice) or passively (e.g., reading what other users post and not engaging in communication). Emotional experiences are often “contagious” in that they can be transmitted from one person to another, which is so-called emotion contagion effects. Parents who use social media may be susceptible to digital emotion contagion (DEC), which involves being affected by other people's emotional expressions online. In social isolation, parents may become prone to either positive or negative DEC, which can affect their anxiety, stress, and burnout. However, very little is known about the ways that DEC affected parents during the COVID-19 pandemic.

In light of its easy transmission and severity of its symptoms, many individuals have experienced anxiety about contracting COVID-19. This can be interpreted as a specific manifestation of illness anxiety, which refers to a set of emotional experiences that are tied to imaginary threats of becoming ill. It is normal and adaptive to have some level of anxiety and concern about one's health (9) because this can motivate protective actions like handwashing and following social distance guidelines. However, individuals who are fearful of a pandemic illness can become excessive and maladaptive, leading to significant distress and impairment in functioning (10). According to Schimmenti (11), the COVID-19 pandemic is characterized by the following fear experiences: (a) fear of the body/fear for the body, (b) fear of significant others/fear for significant others, (c) fear of not knowing/fear of knowing, and (d) fear of taking action/fear of inaction. This set of fears manifests in anxieties that people may have about the virus. One of the core features of these fears and anxieties is personal relevance of the pandemic that increases fear reactions along with the presence of the chronic illness and death in the family due to COVID-19 (4). In the present study, we asked participants if any of their relatives had been infected with COVID-19, aiming to examine the relationship between one of the anxieties mentioned above (i.e., fear for significant others), excessive concern about COVID-19, and parental burnout.

Risk factors for excessive illness anxiety about COVID-19 include heightened trait health anxiety and cyberchondria, while receiving realistic information about the pandemic and using adaptive ER strategies appear to help individuals to cope with anxiety (4). According to Jungmann and Witthöft (4), excessive use of the internet during the pandemic may be considered a safety-seeking behavior that people use to cope with illness anxiety toward COVID-19. However, this intensive internet browsing may paradoxically affect people's emotions as they may read threatening information that increases anxiety.

COVID-19 is highly publicized in the mass and social media. Past research on other pandemic illness outbreaks showed that media reports about the spread of virulent illnesses such as H1N1 “Swine Flu,” Ebola, and Zika led to excessive anxiety and stress (8). Therefore, the extent to which COVID-19 has been covered in the media and online is likely to lead to high levels of anxiety. However, the association between using social media and anxiety is complex and multifaceted. There is evidence of increased life satisfaction as a result of social media usage (12). Simultaneously, Dhir et al. (13) showed that compulsive social media usage evoked social media fatigue, which resulted in elevated anxiety, fear of missing out valuable information, and depression. Research indicated that mothers use social networking sites to seek information about the expectations of motherhood, improve confidence as a mother (14), compare themselves to other mothers, and express emotions (15). Online communication may involve social support and foster a sense of connection and increase well-being when people use Facebook actively (16). Meanwhile, passive usage of social media decreases emotional well-being and increases envy, which, along with social comparison, moderates the relationship between Facebook use and depression (17). Amaro (18) found that greater downward comparison led to greater parenting satisfaction. Thus, if a mother compares herself to other mothers and concludes that she is more successful, she feels satisfied as a parent.

Currently, there is a limited amount of data on the relationship between social media usage by parents, anxiety, and burnout during a Pandemic. These relationships may be moderated by susceptibility to digital emotion contagion (DEC), which is the tendency to mimic and synchronize nonverbal behaviors with those of another person (19). According to Hess and Fisher (20), emotional mimicry evolves in a specific social context when people seek affiliation, thus, most often people aren't mimicking emotions of strangers or antagonistic emotions. However, this relates mostly to positive emotions (e.g., excitement and happiness), while negative emotions (e.g., anger and sadness) are more likely to be contagious among strangers, which forms an emotional ripple effect (21, 22) and amplifies shared stressful experiences (23). Moreover, when people observe others dealing with a stressful situation, their cortisol levels elevate, eliciting affective stress contagion (24). Thus, if a person is surrounded by strangers, they will be more likely to “catch” negative emotions than positive ones. This is especially relevant for the online communication where people are susceptible to DEC (25). DEC is further mediated by social media and other online communication platforms. It can increase both positive (e.g., joy, love, compassion) and negative (e.g., fear, anxiety, sadness) emotions (26). Additionally, social media (e.g., Facebook) may lead to envy and decline in positive mood over time (27). Given that the relationship between DEC and social media is still an evolving topic in the field and that COVID-19 is an emerging situation, little is known about how DEC affects parents who use social media platforms during COVID-19 pandemic. Furthermore, very few studies have examined personal characteristics related to emotion contagion. Goldenberg and Gross (25) argue that the degree of contagion may be affected by social media behaviors, age, gender, culture, and time spent online along with the activity vs. passivity. One of the personal characteristics that leads to emotion contagion may be proneness to engage emotionally with other people online. Doherty (28) developed a measure for emotion contagion to assess individual differences in “susceptibility to emotion contagion (i.e., the likelihood of “catching” the emotions of others)” (p. 132). Scores on this measure indicated that emotion contagion was positively associated with sensitivity to others, self-esteem, and empathy. Susceptibility to catch other people's emotions was negatively associated with self-assertiveness, emotional stability, and alienation (p. 149). Ferrara and Yang (29) identified two types of individuals based on the level of susceptibility to emotion contagions: highly and scarcely susceptible users. Although people of both types are equally prone to take on positive emotions, there are different in the inclination to adopt negative emotions, with scarcely susceptible users having higher negative emotions.

Research on the relationship between burnout and emotion contagion is scarce. Petita and Jiang (30) found a positive relationship between burnout and contagion of fear and a negative relationship between joy contagion and burnout. The authors explored the relationship between job uncertainty and emotion contagion and argued that the contagion of fear increases the feeling of uncertainty, which leads to exhaustion. Uncertainty accompanies parents who are trying to balance work and family during the pandemic, leading to parental burnout.

Parental burnout is a combination of a shattering exhaustion and a feeling that you are not good enough as a parent (6), which often stems from social comparison (31). Precursors to parents becoming burned out may include experiencing high levels of parenting stress, social pressure to be an ideal parent, trying to avoid parenting mistakes, assuming primary responsibilities for caring for the children in comparison to the partner's parenting responsibilities. Mikolajczak and Roskam (6) established a theoretical framework for understanding parental burnout through the perspective of keeping a balance between risks and resources. Risks are defined as factors that increase parental stress, such as low emotional intelligence, lack of support, and excessive parental duties. Resources, on the other hand, decrease stress and enhance well-being. Parental stress may be alleviated by regular self-compassion practices, self-care, social support, and positive co-parenting. During the COVID-19 pandemic outbreak, parental stress levels have increased as the perceived risks increase. Many parents are fearful about becoming ill, not only for themselves but also for their loved ones. Additionally, the burden on many parents has increased as they juggle both working and homeschooling their children. Others have suffered lost jobs or have had to keep going to work in places where they could become infected with the COVID-19. These risks strain typical resources that parents use to maintain the risks/resources equilibrium. Thus, to cope and maintain well-being, a parent needs to add more resources, which is often challenging in the midst of the crisis, leading to the development of the burnout.

According to Roskam et al. (32), parental burnout consists of four components: (a) contrast, (b) saturation, (c) distancing, and (d) exhaustion. Contrast represents the change between what a person used to be as a parent and being ashamed for what they have become as caregivers. Saturation is metaphorically described as being “fed up” with parenting. Distancing is an inability to do anything outside of usual routines. Finally, exhaustion represents overtiredness associated with the parenting role. These four components are of particular interest to the current study. The contrast scale resembles the burnout coming from comparing oneself in the current situation with a parent you were before. Parents may compare themselves with how they used to parent before the COVID-19 pandemic and may feel ashamed for not being good enough parents. Distancing could show itself through inability to change the routine easily, which would be understandable given increased parenting demands. Exhaustion would show that parents do not have enough resources to handle their responsibilities during the pandemic. Saturation would explain how being a parent is not something mothers and fathers enjoy during quarantine.

Burnout is tied to stress, which increases in emergency situations such as COVID-19 pandemic. Various factors can moderate the relationship between stress and burnout, such as social support (33), optimism, pessimism, and coping (34). Etzion (33) found that social support mitigates the effect of stress on burnout. Riolli and Savicki (34) discovered that the lower optimism and higher pessimism were related to depersonalization and emotional exhaustion under high chronic stress, and higher escape coping led to depersonalization. Although these studies were not conducted on parents, they provide insight on the potential relationship between stress and burnout that researchers may find among parents during the COVID-19 pandemic. For instance, Koeske and Koeske (35) found that parental stress was associated with lower role satisfaction and self-esteem among mothers who did not get enough social support, which aligns with the research done by Etzion. Seeking social support may be one of the ER strategies that mothers may use when they try to change their negative emotions such as illness anxiety or depression. Social support can help mothers to get ideas on how to reinterpret the meaning of the situation with ER strategy of cognitive reappraisal. However, little is known about the role of ER in the relationship between stress and parental burnout. This represents an important gap in the literature, given that ER is a powerful resource that could aid in mitigating the stress brought up by COVID-19.

Experiences of stress, burnout and negative emotions during the COVID-19 pandemic take a toll on parents who may try to regulate how they feel using a variety of strategies and techniques to modify emotional experiences and expressions. Emotion regulation (ER) represents time-limited, goal-directed, situationally relevant efforts to change positive and/or negative emotional states. People use ER to uplift and/or down-regulate both positive and negative emotions [(36), p. 1]. ER benefits the self, partners, and family members, alleviating the burden of stress (37). Two most frequently researched ER strategies are cognitive reappraisal and expressive suppression (38). Cognitive reappraisal represents an attempt to change the meaning of the situation that evoked an emotion to change its emotional relevance (38). Expressive suppression defines efforts to hide emotional expression and pretend that the emotion is not taking place. Although, there is evidence showing positive effects of suppression [e.g., (39)], this strategy is found to be less effective than reappraisal because it decreases behavioral expression of an emotion and is less likely to change an emotion experience while increasing physiological reactions for people who suppress emotions (40). Cognitive reappraisal is effective in changing both internal emotional experience and external behavioral manifestation of this experience (41). One of the explanations why cognitive reappraisal may be more effective is that it is usually used when the emotion starts to unfold and is not as strong as it becomes when suppression is used, thus, requiring fewer resources to change the emotion (42). An example of cognitive reappraisal used by a parent during the COVID-19 pandemic could be trying to think about a situation with homeschooling as an opportunity to spend more time with children. An example of emotion suppression would be feeling frustrated with a child who is not doing their homework and trying to hide frustration, pretending that it doesn't affect the parent. Both reappraisal and suppression were found to be related to specific coping strategies. People who often use reappraisal are more satisfied with life, more optimistic, and have greater self-esteem (38). However, it is possible that parents under conditions of high stress may have limited abilities to use cognitive reappraisal (43). In the current study, ER was measured to assess its potential moderating role between concern about COVID-19 and parental burnout.

## Research Hypotheses

The current study provides insight about the mechanisms that moderate the effect of stress on parental burnout, including the potential mitigating function of ER on the development of parental burnout among parents during a Pandemic.

Our hypotheses were:

*H1: Degree of concern about COVID-19 predicts parental burnout*.
*H2: Digital emotion contagion moderates the relationship between concern about COVID-19 and parental burnout*.
*H3: ER moderates the relationship between digital emotion contagion and concern about COVID-19*.

## Materials and Methods

### Procedure

Following institutional review board (IRB) approval, the first author recruited participants on social media (Facebook, Twitter, and Instagram) via poster flyr campaigns, online research system at the Florida International University (SONA), and word of mouth. The researchers also used a snowball sampling approach by asking participants to forward the information about the study to other parents. The researchers used Qualtrics, an online survey portal, to distribute the survey and collect data in April 2020.

### Participants

The targeted population was adults who had children living with them. There were 155 parents who participated in the study, 142 mothers (92%) and 10 fathers (7%). The average age of participants was 37.25 years old (*SD* = 8.20), with an age range of 21–59. Forty percent of participants had one child, 41.3% had two children, and 12.3% had three children, and 0.6% had four children. The average age of a child was 12.6 years old. The majority of the parents were married (78.1%). Over a third of the participants had a doctoral degree (39.4%), 30.3% had associate and 13.5% had a master's degree. The participants were mostly White (73.5%), 12.9% of them identified themselves as Black, and 32.3% were Hispanic/Latino (see Table 1).

**Table 1:** Demographic background of participants.

**Variables**	**Categories**	**Frequency**	**Percent**
Gender	Mother	142	91.6
	Father	10	6.5
# of Children	1	62	40.0
	2	64	41.3
	3	19	12.3
	4	1	0.6
Education	less than high school	3	1.9
	High school or equivalent	1	0.6
	Some college	3	1.9
	Associate degree	47	30.3
	Bachelor's degree	14	9.0
	Master's degree	21	13.5
	Professional/Specialty degree	2	1.3
	Doctoral degree	61	39.4
	Others	1	0.6
Race	Black/African American	20	12.9
	White/Caucasian	114	73.5
	Asian	4	2.6
	Others	16	10.3
Ethnicity	Hispanic/Latino	50	32.3
	Non-Hispanic/Latino	100	64.5

### Measures

#### Concern About COVID-19

Concern about COVID-19 was measured by COVID-19 Threat Scale [CTS; (44)]. The CTS is a self-report inventory that was developed by adapting a questionnaire assessing anxiety in response to the H1N1 “Swine Flu” Influenza (8). Items on the CTS quantify threat-related perceptions of the Coronavirus utilizing a 5-point Likert Scale (from 1- “Not at all” to 5- “Very Much”). Items asked participants to rate their fears that COVID-19 will spread widely in the United States, their fears about becoming ill or family members becoming ill, as well as behavioral changes in response to COVID-19 (e.g., decisions to be around other people, handwashing). Higher scores reflect a greater level of anxiety and more threat-related behaviors due to COVID-19. The Cronbach's Alpha of the scale was 0.84 in the current study.

### Digital Emotion Contagion

Susceptibility for digital emotion contagion was measured via a modified emotion contagion scale developed by Doherty (28). The scale has 15 items and five subscales: happiness, love, fear, anger, and sadness. The items were modified to reflect online communication. An example of an item for the love subscale was, “When I look at the social media pictures of the one I love, my mind is filled with thoughts of romance.” An example of an item for the happiness subscale was, “Being with a happy person on social media picks me up when I'm feeling down.” The sadness scale had items such as, “I cry at sad videos on social media.” An example of an item for the fear subscale was, “Watching the fearful faces of victims on the news makes me try to imagine how they might be feeling. The Anger subscale was represented by items such as, “I clench my jaws and my shoulders get tight when I see the angry faces on the news on social media.” Participants rated their responses on a five-item Likert-type scale (from 5— “Always” to 1— “Never”). Cronbach's Alpha for the susceptibility for the digital contagion scale was 0.86 in the current study.

### Parental Burnout Assessment

Parental burnout was measured using the 23-item Parental Burnout Assessment (PBA; 24). The PBA is used to assess the levels of exhaustion, saturation, contrast, and distancing of parental burnout. Sample items include “I find it exhausting just thinking of everything I have to do for my child(ren),” (exhaustion subscale), “I feel like I can't take any more as a parent” (saturation subscale), “I'm ashamed of the parent that I've become” (contrast subscale), and “I'm no longer able to show my child(ren) how much I love them” (distancing subscale). Participants rated items on a 7-point Likert scale with response options ranging from “Never” to “Every day.” High scores reflect a high level of parental burnout. Cronbach's alpha of the total scale was 0.97 in the current study. Alphas of items measuring exhaustion, contrast, saturation, and distancing were 0.95, 0.92, 0.95, and 0.86, respectively.

### Depression, Anxiety and Stress Scale (DASS-21)

Distress was measured using Depression, Anxiety and Stress Scale (DASS-21; 35), which included three 7-item subscales (depression, anxiety and stress). Examples of items were, “I couldn't seem to experience any positive feeling at all” (depression subscale), “I found it difficult to relax” (stress subscale) and, “I was worried about situations in which I might panic and make a fool of myself” (anxiety subscale). Participants rated their responses on a 4-item scale, where 0 was “Did not apply to me at all,” 1— “Applied to me to some degree, or some of the time,” 2— “Applied to me to a considerable degree or a good part of time,” and 3—Applied to me very much or most of the time.” In the current study, Cronbach's alpha of the total scale was 0.94. Alphas of items measuring stress, anxiety, and depression were 0.90, 0.82, and 0.87, respectively.

### Emotion Regulation

Emotion Regulation Questionnaire [ERQ; (38)] was used to assess participants' tendency of using cognitive reappraisal and/or expression suppression to regulate their emotions. The items were rated on a 7-point Likert-type scale (1 = “Strongly disagree” to 7 = “Strongly agree”). An item example of the cognitive reappraisal subscale was, “When I want to feel more *positive* emotion (such as joy or amusement), I *change what I'm thinking about*.” An item example for the expressive suppression subscale was, “When I am feeling *positive* emotions, I am careful not to express them.” Cronbach's Alpha of the ERQ was 0.82.

## Data Analysis

Before main data analyses were performed, we checked the effects of participants' gender and age on the main variables we examined. Concern about COVID-19 was not significantly different across gender (*t*^1^_(9.31)_ = 0.11, *p* = 0.91). Though significant differences were found among different age groups [*F*_(3, 68.9 = 08)_ = 4.72, *p* = 0.005], *post-hoc* analysis showed that there was only a marginally significant difference between 21–30 and 41–50 age groups (*t* = 0.52, *p* = 0.05). Digital emotional contagion among parents was not significantly different across gender (*t*_(9.33)_ = 0.80, *p* = 40) and age groups [*F*_(3, 149 = 08)_ = 0.85, *p* = 0.47]. These were the same with total score of parental burnout (gender, *t*_(9.61)_ = 0.18, *p* = 0.86; age groups, *F*_(3, 149)_ = 2.27, *p* = 0.08) and emotional regulation questionnaire scores (gender, *t*_(9.28)_ = 0.50, *p* = 0.63; age groups, *F*_(3, 45.03)_ = 1.39, *p* = 0.26). DASS score was not significant across gender (*t*_(9.54)_ = 0.45, *p* = 0.66). However, it was significantly different across age groups [*F*_(3, 49.76)_ = 9.28, *p* < 0.001] and parents of 31–40 years old had significantly higher score than those over 50 years old (*p* = 0.001). Further analyses found that stress [*F*_(3, 149)_ = 4.56, *p* = 0.004] and depression scores were significantly different across age groups [*F*_(3, 149)_ = 2.93, *p* = 0.04], but anxiety was not significant [*F*_(3, 149)_ = 1.84, *p* = 0.14]. These indicated that gender was not a confounding variable in the main analyses but age was one when stress and depression were the dependent variables.

Correlational analysis was first used to examine the relationships between concern about COVID-19, stress, DEC, general anxiety and depression, parental burnout, and ER. Linear and multiple linear regression analysis was conducted to assess the relationships between illness anxiety, parental burnout, DEC, and ER. Moderation analyses were performed to address hypotheses two and three. Moderation focuses on “when” questions and a moderation effect is usually present when a third variable affects the relationship between predictor and outcome variables (45). All the analyses were conducted in IBM SPSS (Version 26), and moderation analysis was conducted via PROCESS SPSS—a versatile modeling tool developed to integrate many features that exist separately in a few popular statistical software programs, such as, mean centering predictors to reduce multicollinearity (46), providing information about how much variance in the outcome variable can be explained by the model and specifically by the interaction (47). Additionally, participants were asked questions regarding their personal experiences during COVID-19 Pandemic, such as: “Do you personally know anyone who got infected with Coronavirus?” and “Do you personally have a relative who is currently infected with Coronavirus? They were also asked to report their demographic background information, such as age, gender, race, ethnicity.

## Results

Correlations among study measures are presented in Table 2. Linear regression analysis then examined whether concern about COVID-19 predicted stress and depression. Because age was had a significant effect on stress and depression, it was added to the regression models (see Table 3). The results showed that both stress and depression were predicted by concern about COVID-19 [*F*_(2, 150)_ = 10.80, *p* < 0.001 for stress; *F*_(2, 150)_ = 6.00, *p* = 0.003 for depression]. However, age was not significant in both models (*p* = 0.50 for stress; *p* = 0.94 for depression). In turn, Concern about COVID-19 moderately predicted parental burnout, *F*_(1, 154)_ = 5.465, *p* < 0.05, which supports H1. Comparatively speaking, concern about COVID-19 explained more variance in stress (12.6%, β = 0.36, *t* = 4.64, *p* < 0.001) than in depression (7.4%, β = 0.27, *t* = 3.37, *p* = 0.001) and parental burnout (3.4%, β = 0.19, *t* = 2.34, *p* = 0.02) (see Table 2).

**Table 2:** Descriptive statistics and correlations for study variables.

**Variable**	***M***	***SD***	**1**	**2**	**3**	**4**	**5**	**6**	**7**	**8**
1. Depression	1.8	0.71	1	0.80^**^	0.59^**^	0.55^**^	0.59^**^	0.60^**^	0.27^**^	0.20^*^
2. Stress	2.3	0.8	0.80^**^	1	0.62^**^	0.49^**^	0.48^**^	0.43^**^	0.25^**^	0.225^*^
3. PBA Exhaustion	3.2	1.8	0.58^**^	0.62^**^	1	0.79^**^	0.76^**^	0.64^**^	0.24^**^	0.20^*^
4. PBA Contrast	2.2	1.5	0.55^**^	0.49^**^	0.79^**^	1	0.84^**^	0.79^**^	0.09	0.25^**^
5. PBA Saturation	2	1.5	0.59^**^	0.48^**^	0.76^**^	0.84^**^	1	0.84^**^	0.14	0.17^*^
6. PBA Distancing	1.8	1.3	0.60^**^	0.43^**^	0.64^**^	0.79^**^	0.85^**^	1	0.12	0.18^*^
7. Concern about COVID-19	4.2	0.63	0.27^**^	0.35^**^	0.24^**^	0.09	0.14	0.12	1	0.27^**^
8. Digital Emotion Contagion	3.3	0.66	0.20^*^	0.22^**^	0.20^*^	0.25^**^	0.17^*^	0.17^*^	0.27^**^	1

* *p < 0.05*.

** *p < 0.01, N = 155*.

**Table 3:** Regression analyses of concern about COVID-19 and stress, depression, and parental burnout.

**Predictor**	**B**	**SE**	**β**	***t***	***p***
**Regression Analysis of Concern about COVID-19 and Stress**					
(Constant)	0.46	0.42		1.01	0.27
Concern	0.46	0.10	0.36	4.64	0.00
Age	−0.05	0.07	−0.05	−0.68	0.50
*R^2^ =0.121*					
**Regression Analysis of Concern about COVID-19 and Depression**					
(Constant)	0.48	0.38		1.26	0.21
Concern	0.31	0.09	0.27	3.37	0.001
Age	0.01	0.07	0.01	0.07	0.94
*R^2^ =0.074*					
**Regression Analysis of Concern about COVID-19 and Depression**					
(Constant)	0.68	0.78		0.87	0.39
Concern	0.43	0.19	0.19	2.34	0.02
*R^2^ =0.034*					

The moderation effect of digital emotion contagion (DEC) on the relationship between concern about COVID-19 and total score of the parental burnout measure was not significant, thus, H2 was not supported. However, analysis of the subscales of parental burnout yielded significant results. More specifically, the moderation effect of DEC on the relationship between stress and parental contrast sub-scale showed that both stress (*p* < 0.001) and DEC (*p* = 0.02) and the interaction between stress and DEC (*p* = 0.04) significantly predicted contrast and all were positive predictors (see Table 4). This suggests that stress has a significant effect on parental contrast when emotion contagion is high, or in other words, emotion contagion significantly increases the impact of stress on parental contrast. The whole model explains 28% of the variance in parental contrast, with about 2% contributing from the interaction. Comparatively speaking, stress had a higher coefficient, while DEC and the interaction had very similar coefficients (see Table 4).

**Table 4:** Moderation analyses of DEC between stress and parental burnout sub-scales.

**Predictor**	**B**	**b 95% CI [LL, UL]**	**SE**	***t***	***p***
**Moderation Effect of DEC between stress and parental contrast**					
(Constant)	2.15	1.95–2.35	0.10	21.05	<0.001
Stress	0.79	0.53–1.05	0.13	6.05	<0.001
Emotion contagion	0.37	0.06–0.68	0.16	2.33	0.02
Interaction	0.35	0.02–0.69	0.17	2.09	0.04
*R*^2^ =0.28					
**Moderation Effect of DEC between stress and parental exhaustion**					
(Constant)	3.17	2.94–3.40	0.12	27.24	<0.001
Stress	1.35	1.06–1.64	0.15	9.09	<0.001
Emotion contagion	0.17	−0.19–0.52	0.18	0.94	0.35
Interaction	−0.02	−0.40–0.36	0.19	−0.09	0.93
*R*^2^ =0.38					
**Moderation Effect of DEC between stress and parental saturation**					
(Constant)	1.95	1.74–2.17	0.11	17.82	<0.001
Stress	0.86	0.58–1.13	0.14	6.13	<0.001
Emotion contagion	0.20	−0.14–0.53	0.17	1.17	0.24
Interaction	0.34	−0.02–0.70	0.18	1.87	0.06
*R*^2^ =0.26					
**Moderation Effect of DEC between stress and parental distancing**					
(Constant)	1.74	1.54–1.93	0.10	17.72	<0.001
Stress	0.65	0.40–0.89	0.12	5.19	<0.001
Emotion contagion	0.22	−0.08–0.52	0.15	1.44	0.15
Interaction	0.41	0.09–0.73	0.16	2.52	0.01
*R^2^* = 0.23.					

The same analyses were conducted with the other three subscales of parental burnout. Stress significantly predicted parental exhaustion (*p* < 0.001) and saturation (*p* < 0.001), but DEC did not (*p* = 0.35 for exhaustion, *p* = 0.24 for saturation) and the interaction terms were also not significant (*p* = 0.93 for interaction in exhaustion model, *p* = 0.06 for saturation model). In addition, stress significantly predicted parental distancing (*p* < 0.001), while emotion contagion did not (*p* = 0.15). However, the interaction of stress and DEC was a significant predictor of parental distancing (*p* = 0.01) (see Table 4). This indicates that, like with the parental contrast subscale, emotion contagion increases the impact of stress on parental distancing. The whole model explained 23% of the variance in parental contrast, with about 3% variance accounted for by the interaction.

The effect of stress on parental burnout was further moderated by knowing people who are infected with COVID-19 (*p* < 0.001). Stress (*p* < 0.001) and the interaction between stress and knowing people infested with COVID-19 (*p* < 0.01) had significant positive effects on parental burnout, indicating that having relatives infected with COVID-19 increased the effect of stress on parental burnout. The whole model explained 37% of the variance in parental burnout. The effect of DEC on parental burnout was also moderated by having relatives infected with COVID-19. DEC (*p* < 0.01) and the interaction (*p* < 0.001) between DEC and having a relative infected with COVID-19 had significant positive effects on parental burnout, indicating that having relatives infected with COVID-19 increases the effect of DEC on parental burnout. The whole model explained 15% of the variance in parental burnout (see Tables 5, 6).

**Table 5:** Moderation effect of having relatives infected with COVID-19 between parental burnout and DEC.

**Predictor**	**B**	**b 95% CI [LL, UL]**	**SE**	***t***	***p***
Constant	2.48	2.27–2.70	0.11	22.67	<0.001
Digital emotion contagion	0.43	0.10–0.76	0.17	2.57	0.01
Having relatives infected with COVID-19	0.40	−0.32–1.11	0.36	1.10	0.27
Interaction	1.96	0.99–2.92	0.49	4.02	<0.001
*R^2^* = 0.15.					

**Table 6:** Moderation effect of emotion regulation between DEC and concern about COVID-19.

**Predictor**	**B**	**b 95% CI [LL, UL]**	**SE**	***t***	***p***
(Constant)	4.19	4.10–4.29	0.05	90.09	<0.001
Emotion contagion	0.24	0.10–0.38	0.07	3.32	<0.001
Emotion regulation	−0.05	−0.14–0.04	0.05	−1.07	0.29
Interaction	−0.20	−0.30 to −0.11	0.05	−4.17	<0.001
*R*^2^ =0.18.					

We also tested *H3* through moderation analysis. The results indicated that DEC significantly predicted concern about COVID-19 (*p* < 0.001), whereas individual ER did not (*p* = 0.29). The interaction between DEC and ER had a significant (*p* < 0.001) yet negative effect on the relationship (see Table 7). This indicates that a higher level of ER significantly reduces the effect of DEC on concern about COVID-19, suggesting a beneficial effect of ER. The full model explains 18% of the variance in parental contrast, with about 10% accounted for by the interaction term.

**Table 7:** Moderation effect of having relatives infected with COVID-19 between parental burnout and stress.

**Predictor**	**B**	**b 95% CI [LL, UL]**	**SE**	***t***	***p***
(Constant)	2.45	2.26–2.63	0.09	25.88	<0.001
Stress	1.05	0.82–1.29	0.12	8.88	<0.001
Having relatives infected with COVID-19	−0.30	−0.94–0.35	0.33	−0.91	0.36
Interaction	0.98	0.26–1.70	0.36	2.70	0.01
*R^2^* =0.37.					

## Discussion

This study showed that concern about COVID-19 predicted stress, depression, and parental burnout. These results align with findings of Koutsimani et al. (5), who found that people experiencing higher levels of anxiety are more prone to burnout. Likewise, we found parents who experienced higher levels of anxiety in response to COVID-19 and who believed they had higher possibility of being infected tended to experience higher levels of parental burnout in all four domains.

Anxiety is accompanied with intrusive thoughts and may lead to mental fatigue, becoming a risk factor that increases parental stress disturbing the balance between risks and resources described by the theoretical framework of parental burnout developed by Mikolajczak and Roskam (6). Thus, parents who experience higher levels of concern about the virus might benefit from using resources such as self-compassion practices and social support to decrease anxiety and stress. This is especially true if they have relatives infected with COVID-19 because the virus becomes more personal to them, which may increase their illness anxiety (4).

Having relatives infected with COVID-19 increased the effect of stress on parental burnout along with the effect of DEC on parental burnout. It is possible that the personal experience of having the virus affect someone you know increases the anxiety about the COVID-19, which becomes an additional risk factor for parents exhausting their coping resources. The increased DEC effect on parental burnout when a parent has a relative infected with COVID-19 could be due to that a person may engage in excessive use of internet during pandemic. This is considering a safety-seeking behavior that people use to cope with illness anxiety (4). However, actively searching for virus-related information may become a risk factor for parents as it may trigger negative emotions due to DEC.

The current research showed that parental burnout is affected by susceptibility to emotion contagion. More specifically, DEC increased the impact of stress on two subscales of parental burnout: contrast and distancing. During quarantine in a Pandemic, ways to obtain social support are often limited to digital communication. This result means that the more parents are susceptible to take on other people's emotions on social media, the more they tend to feel that they are not as good parents as they used to be and that they are no longer able to make efforts for their children and can't do anything out of usual routines as parents. One of the explanations for the moderating role of DEC on the relationship between stress and contrast is that pandemic changed lives of parents. While completing the survey, they may, consciously or unconsciously, have compared pandemic parenting with pre-pandemic parenting and felt that they were not coping well enough during this crisis. De los Santos et al. (15) found that mothers on social media tend to express negative emotions more often than positive, thus, susceptibility to DEC of negative emotions makes a mother experience more anger, sadness, and fear herself. Regarding burnout related to perceived contrast, mothers may seek information about the expectations of motherhood and try to improve their confidence as mothers (14). However, when they compare themselves to other mothers and see that they are not doing as well as other parents, they may feel ashamed and think about their pre-pandemic parenting, which was different and up to their standards of a “good mother.”

Distancing-related burnout involves difficulty doing anything out of standard routines, and it may be exacerbated by social media discussions between mothers when they express negative emotions about new responsibilities they need to fulfill in pandemic parenting. For instance, in Facebook mothers' groups, parents emotionally discuss how tired they are and how they are not planning to do extra work as parents because they have no energy to do so. DEC may then make other parents experience the same emotions. Paradoxically, these results may show that social media does foster a sense of connection among parents during the Pandemic, but this connection is not making them feel better, rather it leads to burnout through DEC. It may happen because negative emotions in general are more contagious among strangers (21, 22), amplifying shared stressful experiences (23). Thus, when mothers on social media observe other mothers' struggles, they feel more stressed themselves through affective stress contagion (24). However, we do not know if participants used social media actively or passively. Passive use is more likely to be problematic, as Appel (17) showed passive usage decreases well-being and increases envy, leading to depression.

The results showed that a higher level of ER reduced the relationship between digital emotion contagion and concern about COVID-19, thus ER may have a positive effect on this relationship. This means that when parents use ER strategies after they become emotionally involved in DEC on social media, they tend to have lower anxiety about COVID-19. This result aligns with the research on cognitive reappraisal as an ER strategy. Parents could use cognitive reappraisal to change the meaning of the situation and improve their emotional experiences (38). For example, when a parent is navigating social media websites and starts feeling anxious about COVID-19 because other users are expressing fears, they could try to re-interpret the situation and/or change its meaning. An example of a cognitive reappraisal would be, instead of thinking, “this virus is going to kill me,” they think, “I am using all the necessary precautions and the chance to get infected for me or my family is low.” This relationship may reflect the awareness of DEC among study participants. They may have recognized that being on social media may make them prone to take on other people's emotions and, thus, they may have tried to regulate their feelings and decrease anxiety.

## Limitations and Future Research

An important limitation of the present study is the use of a cross-sectional design, in which participants completed the measures only once. Thus, we are unable to draw cause and effect relationships among the study variables. Future research should utilize longitudinal measures to study the relationships among anxiety, internet use, ER, and parental burnout. Another limitation is the limited size and diversity of the sample. We also did not include questions regarding parental communication in social media groups, and didn't ask how much time participants spend looking for information about COVID-19, these questions could have informed present research and would have made our discussion of findings more robust.

Future studies may focus on socio-demographic differences between parents of different races, ethnicities, and marital status. Additionally, researchers may want to investigate fathers' emotional experiences during the Pandemic and compare those with maternal feelings and behaviors. Another limitation is that only cognitive reappraisal and expressive suppression were assessed. Future projects may focus on the difficulties in ER, assessing broader range of ER strategies and techniques that parents use to cope with concern about COVID and burnout.

Qualitative interviews with parents exploring ways that they use social media could help to obtain an in-depth understanding of the function of social media in parental burnout, concern about COVID-19, and emotion regulation during pandemic. One of the factors that impacts DEC is activity vs. passivity of a social media user. Future studies could investigate the relationship between activity and/or passivity on social media, the type of social media, DEC, ER, and burnout among parents.

## Conclusion

The present study explored the role of susceptibility to digital emotion contagion in the relationship between concern about COVID-19, stress, parental burnout, and emotion regulation. We found that parents who were more susceptible to digital emotion contagion were experiencing higher parental burnout when feeling stressed. Parents who used emotion regulation strategies when they experienced emotion contagion had lower anxiety about COVID-19. These data suggest that digital emotion contagion media affects experiences of stress and burnout in parents and that emotion regulation helps to mitigate these effects during a pandemic threat.

## Data Availability Statement

The raw data supporting the conclusions of this article will be made available by the authors, without undue reservation.

## Ethics Statement

The studies involving human participants were reviewed and approved by the Social and Behavioral Institutional Review Board of Florida International University. The patients/participants provided their written informed consent to participate in this study.

## Author Contributions

AP developed and designed the methodology, conducted data collection, applied statistical techniques to analyze and synthesize the study data, prepared the published work, specifically writing the initial draft. HL applied statistical techniques to analyze and synthesize the study data, provided the analysis tool and prepared the published work, specifically with critical reviews, editing, and revisions. MW assisted in developing the research plan and data collection, and prepared the published work, specifically with critical reviews, editing and revisions. All authors contributed to the article and approved the submitted version.

## Conflict of Interest

The authors declare that the research was conducted in the absence of any commercial or financial relationships that could be construed as a potential conflict of interest.

## Footnotes

^1^When statistical assumption of homogeneity of variance was not supported in the independent sample t-test, the degree of freedom was a decimal to correct for the non-homogeneity of variance. When the same assumption was not supported in ANOVA, Welch test was used and a degree of freedom with a decimal was reported for the same purpose.
